# A Herbal Mixture from Propolis, Pomegranate, and Grape Pomace Endowed with Anti-Inflammatory Activity in an In Vivo Rheumatoid Arthritis Model

**DOI:** 10.3390/molecules25092255

**Published:** 2020-05-11

**Authors:** Valentina Parisi, Antonio Vassallo, Claudio Pisano, Giacomo Signorino, Francesco Cardile, Milena Sorrentino, Fabiana Colelli, Alessandra Fucci, Egildo Luca D’Andrea, Nunziatina De Tommasi, Alessandra Braca, Marinella De Leo

**Affiliations:** 1 Dipartimento di Farmacia, Università degli Studi di Salerno, 84084 Fisciano (SA), Italy; v.parisi15@studenti.unisa.it; 2 Università degli Studi di Salerno, Ph. D. School of Pharmacy, 84084 Fisciano (SA), Italy; 3 Dipartimento di Scienze, Università della Basilicata, 85100 Potenza, Italy; antonio.vassallo@unibas.it; 4 Biogem, Research Institute “G. Salvatore”, 83031 Ariano Irpino (AV), Italy; giacomo.signorino@biogem.it (G.S.); francesco.cardile@biogem.it (F.C.); milena.sorrentino@biogem.it (M.S.); fabiana.colelli@biogem.it (F.C.); alessandra.fucci@biogem.it (A.F.); egildoluca.dandrea@biogem.it (E.L.D.); 5 Dipartimento di Farmacia, Università di Pisa, 56126 Pisa, Italy; alessandra.braca@unipi.it (A.B.); marinella.deleo@unipi.it (M.D.L.); 6 Centro Interdipartimentale di Ricerca Nutraceutica e Alimentazione per la Salute “Nutrafood”, Università di Pisa, 56124 Pisa, Italy

**Keywords:** propolis, pomegranate, Aglianico grape pomace, herbal preparation, LC-ESI-MS/MS, rheumatoid arthritis, in vivo test

## Abstract

Rheumatoid arthritis (RA) is a chronic inflammatory autoimmune disease characterized by the production of inflammatory factors. In order to overcome the side effects of currently used anti-inflammatory drugs, several attempts have been made to identify natural products capable of relieving RA symptoms. In this work, a herbal preparation consisting of propolis, pomegranate peel, and Aglianico grape pomace (PPP) extracts (4:1:1) was designed and evaluated for its effect on a murine collagen-induced arthritis (CIA) model. Firstly, the chemical contents of four different Italian propolis collected in the Campania region (Italy) were here reported for the first time. LC-MS analyses showed the presence of 38 constituents, identified in all propolis extracts, belonging to flavonoids and phenolic acids classes. The Pietradefusi extract was the richest one and thus was selected to design the PPP preparation for the in vivo assay. Our results highlight the impact of PPP on RA onset and progression. By using in vivo CIA models, the treatment with PPP resulted in a delayed onset of the disease and alleviated the severity of the clinical symptoms. Furthermore, we demonstrated that early PPP treatment was associated with a reduction in serum levels of IL-17, IL-1b, and IL-17–triggering cytokines.

## 1. Introduction

Rheumatoid arthritis (RA) is an autoimmune disorder affecting about 1% of the global population, mainly women. Both genetic and environmental factors are involved in the development of this disease [1]. Clinically, RA manifests itself as a systemic, chronic inflammatory disease characterized by synovial inflammation and the erosion of bone and cartilage, which leads to the destruction of the affected joints, having an important impact on the individual quality of life in patients and also on social and economic aspects [2,3]. The cause of RA is unknown, but several studies have suggested the involvement of different molecular mechanisms. First of all, being an inflammatory disease, it involves several inflammatory cytokines, such as tumor necrosis factor-α (TNF-α), interleukin-1 (IL-1), interleukin-6 (IL-6), and interleukin-17 (IL-17), which are overexpressed in RA joints and play an important role in its pathogenesis [4,5]. RA treatment involves several approaches, including drugs of both synthetic and biological origins, causing different severe side effects ranging from osteoporosis to liver fibrosis and myocardial infarction; some of them also have quite an expensive price. Owing to these limitations, an increasing number of patients have started to turn to natural products to relieve the symptoms of RA [6,7]. Moreover, oxidative stress is considered to be involved in the pathogenesis of different diseases, such as atherosclerosis, cancer, neurodegenerative disorders, and autoimmune rheumatological diseases, including RA [8]. In this regard, research into natural product-based preparations with potential applications in RA could be important, with particular regard to the combination of two or more active products, which, acting through different mechanisms, may have an effect against the molecular mechanisms involved in RA, thus potentiating their efficacy and benefits [9]. Among the natural products, propolis, pomegranate (*Punica granatum* L., Punicaceae), and grape (*Vitis vinifera* L., Vitaceae) extracts have been subjected to an extensive scientific interest for their possible use in various pathologies, including those of inflammatory origin. In fact, their main components, consisting mainly of phenolic acids, flavonoids, and stilbenes, are promising candidates as therapeutic alternatives in the treatment of RA due to their strong anti-inflammatory activity reported in many in vitro and in vivo studies. The phenol content of both pomegranate and grape strongly depend on the cultivar and variety, as well as the climate and ecological conditions [10,11]. Several studies have reported that pomegranate peels from different geographic areas, including Italian regions, are rich in hydrolyzable tannins, with punicalagin as the main constituent [12]. The phenol content of red grape is characterized by phenolic acids, flavonols, anthocyanins, stilbenes, and condensed tannins, whose amount also remains high in grape pomace (winemaking byproduct) after the fermentation process [13]. These findings were also confirmed in Italian cultivars [14,15].

Propolis is one of the *Apis mellifera* L. elaborations obtained by the recollection of the exudate from different plant species. It is a complex mixture containing resin, balsam, waxes, volatiles, pollen, and, in a small proportion, secondary metabolites (5–10%). Among the last, polyphenols, including flavonoids; phenolic acids and their esters; benzophenones; and lignans are the main components [16]. The propolis effective chemical composition as well as the type and number of bioactive compounds has been reported to be strongly dependent on and influenced by the source of the plant resin, seasons, and geographical origin. This mixture, used by honeybees as a defense against different predators, is nowadays recognized for its antimicrobial, antiviral, antioxidant, immunomodulatory, hepatoprotective, and anti-inflammatory activities, inhibiting the production of IL-17 and the differentiation of Th17 cells [17,18].

Through various different studies, it was proved that the proanthocyanidin-rich grape seed extract provides benefits against many diseases, i.e., inflammation, cardiovascular disease, hypertension, diabetes, cancer, peptic ulcers, and microbial infections [19,20]. A recent combination of two polyphenol-rich extracts, grape and propolis, potentiated anti-inflammatory activity in rats, reducing clinical scores with respect to the corticoid-treated group [21]. Moreover, several studies examined the effects of different pomegranate-related products, including extracts from peel, showing their large spectrum of anti-inflammatory activity [22,23]. Finally, pomegranate extract’s administration in an RA model was shown to reduce oxidative stress, inhibit the p38-mitogen-activated protein kinase (p38-MAPK) pathway, and activate the transcription factor NF-κB [24].

In this context, Campania region (South Italy) folk medicine presents several uses of propolis. In particular, a traditional rural preparation of the internal area (Benevento and Avellino provinces) containing propolis and grape pomace was used in the past in the treatment of inflammation diseases such as body pain and oral inflammation. Based on this evidence, we can suppose that propolis, grape pomace, and *P. granatum* extracts could synergistically contribute to beneficial effects in the RA condition. This assumption is based on the fact that these extracts contain a variety of chemical compounds, including flavonoids, cinnamic acid derivatives, prenylated phenylpropanoids, and both ellagic and gallagic derivatives, showing immunomodulatory and anti-inflammatory effects and inhibiting some inflammatory modulator responses [2].

Thus, a traditional herbal preparation consisting of propolis, pomegranate peel, and grape pomace extracts (4:1:1) was concocted. The grape pomace was obtained from Aglianico, an ancient cultivar cultivated in southern Italy that was found to be very rich in phenol content [25]. The objective of this work was to perform a deep quali-quantitative chemical characterization of propolis coming from four different internal areas of the Campania region (Italy) and to evaluate the preventive and therapeutic efficacy of a natural product-based formulation containing propolis, pomegranate peel, and Aglianico grape pomace on a murine collagen-induced arthritis (CIA) model.

## 2. Results and Discussion

### 2.1. Chemical Characterization of Extracts

#### 2.1.1. Comparative Analysis of Four Different Italian Propolis

**Figure 1:**
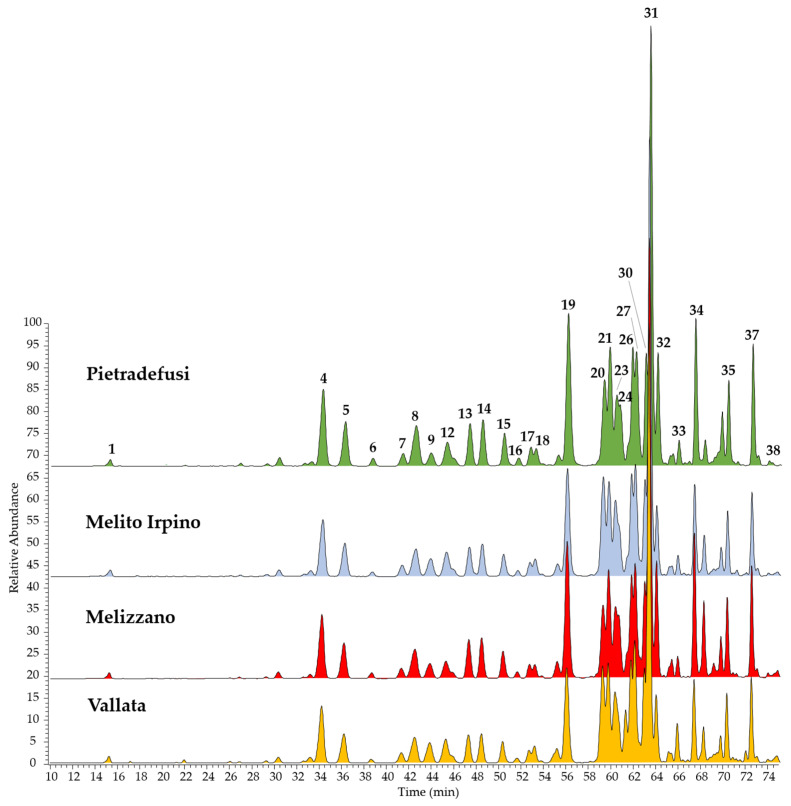
LC-ESI-MS/MS chromatograms, registered in negative ion mode, of four extracts of Italian propolis collected in the Campania region. Peak data are shown in Table 1.

**Figure 2:**
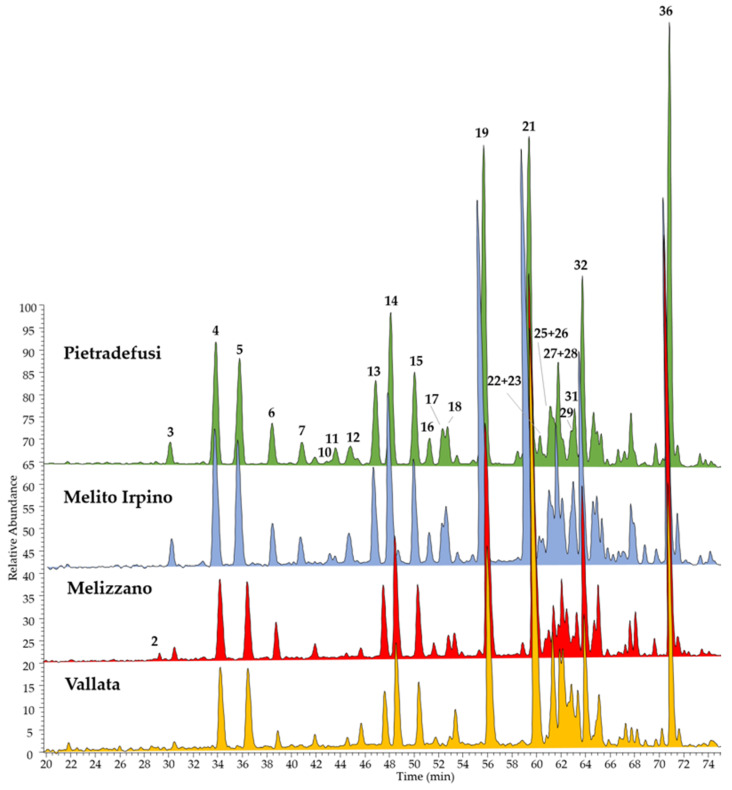
LC-ESI-MS/MS chromatograms, registered in positive ion mode, of four extracts of Italian propolis collected in Campania Region. Peak data are shown in Table 2.

The chemical contents of four different Italian propolis collected from hives located in the Campania region (the rural area of Pietradefusi, Melito Irpino, Melizzano, and Vallata) were herein established by HPLC coupled with a photodiode array (PDA)/UV and an electrospray ionization (ESI) mass spectrometer (MS) for the first time. ESI-MS chromatograms were registered in both negative (Figure 1) and positive ion modes (Figure 2), since propolis is a complex mixture containing different classes of compounds that can be detected using different techniques. In particular, the negative ion ESI-MS mode was suitable for the detection of phenolic acids and their esters, together with esters of pinobanksin.

In total, 38 constituents were identified in almost all propolis extracts, belonging to flavonoids and phenolic acids. The identification of all compounds was carried out comparing elution order, UV absorptions, and both full and fragmentation ESI-MS data of all detected molecules with those reported in previous studies [26,27,28]. Alpinetin, caffeic acid prenyl ester, luteolin, pinobanksin 5-methyl ether, pinobanksin 3-*O*-acetate, and quercetin were confirmed using reference standards. All these data are listed in Table 1 (ESI negative ion mode) and Table 2 (ESI positive ion mode).

Results of the LC-MS analyses showed that, with regard to the qualitative aspect, all the analyzed propolis extracts had very similar chemical profiles, with few differences about the distribution of some metabolites in the various samples. Flavonoids were found to be present in the form of aglycones, as well as expected in propolis since the glycoside forms are hydrolyzed by bees’ glycosidase salivary enzymes [28]. In particular, the following classes of aglycones were found: flavones including chrysin, apigenin, luteolin, and galangin derivatives (compounds **2**, **6**, **7**, **13**, **15**, **17**, **21**, **27**, **28**, **32**, and **36**); flavanones such as alpinetin (**11**) and pinocembrin (**26**); flavonols represented by quercetin, kaempferol, and isorhamnetin derivatives (**3**, **5**, **9**, **12**, **14**, **18**, **19**, and **29**); dihydroflavonols including pinobanksin and their esters (**4**, **8**, **10**, **16**, **31**, **34**, **35**, **37**, and **38**). Among phenolic acids, caffeic acid (**1**) and caffeic acid esters (**20**, **23**, **24**, **30**, and **33**) were found as constituents in all propolis extracts. Compared to previous analyses of Italian propolis collected in different regions, such as Emilia Romagna [29] and Veneto [28], the chemical composition of Campanian propolis was found to be similar particularly in flavonoid content, whereas among phenolic acids, both ferulic and cinnamic acid derivatives were not found.

Quantitative analyses evidenced that the total phenol content in the studied propolis extracts was in the range of 15.54–39.18% (Table 3), with Pietradefusi extract the richest one. In contrast, the propolis collected at Vallata presented a minor content in terms of total phenols, but it was the only extract in which the total phenolic acids were higher than the total flavonoids. Taking into account that phenolic acids were represented by six compounds, whereas quantified flavonoids were 30 different compounds, the estimated amount of caffeic acid derivatives in the propolis is very relevant, with caffeic acid phenylethyl ester (CAPE, compound **30**) and caffeic acid prenyl esters (**20** and **24**) the most representative. Among flavonoids, chrysin (**21**) and chrysin 5-methyl ether (**36**) were the most abundant in all extracts. Other main flavonoids were represented by quercetin dimethyl ether (**19**); galangin 5-methyl ether (**15**); pinobanksin (**8**); and pinobanksin esters such as pinobanksin 3-*O*-acetate (**31**) and pinobanksin 3-*O*-hexanoate (**38**). These results are in agreement with previous studies on Italian propolis that showed a very similar profile, with variability in total phenol content among the different samples [27].

Pietradefusi propolis extract was the richest one and was thus selected to design the herbal preparation PPP for the in vivo assay.

#### 2.1.2. Chemical Content of Pomegranate Peel and Grape Pomace

The pomegranate peel and grape pomace extracts used in the phytopreparation were characterized by LC-MS/MS analyses. All the compounds were tentatively identified by the comparison of spectral and chromatographic data with those of previous studies. According to the literature [30], the main constituents of pomegranate peel were established to be derivatives of ellagic acid (ellagitannins) and gallagic acid (gallagyl esters) (Table 4). Ellagic acid (λ_max_ at 254 and 368 nm) showed a deprotonated molecule at [M − H]^−^ at *m/z* 301; accordingly, in the MS/MS experiments on the detected ellagitannins, a base ion peak at *m/z* 301 was observed. Most of the compounds were in glycoside form, as deduced by the loss of hexosyl (162 u) or pentosyl (132 u) units. The gallagyl derivatives were tentatively identified as punicalin ([M − H]^−^ at *m/z* 781) and punicalagin ([M − 2H]^2−^ at *m/z* 541), two gallagyl esters previously found to be the main components in pomegranate and occurring in the form of two isomers [31].

Grape pomace was found to be very rich in the phenolic compounds such as resveratrol, flavonols (quercetin, laricitrin, syringetin, and myricetin glucosides), many anthocyanins (cyanidin, delphinidin, malvidin, and petunidin derivatives), and flavan-3-ols (catechin/epicatechin and their procyanidin oligomers), in agreement with previous studies (Table 5) [32,33,34,35,36].

### 2.2. Murine RA Model Assay

**Figure 3:**
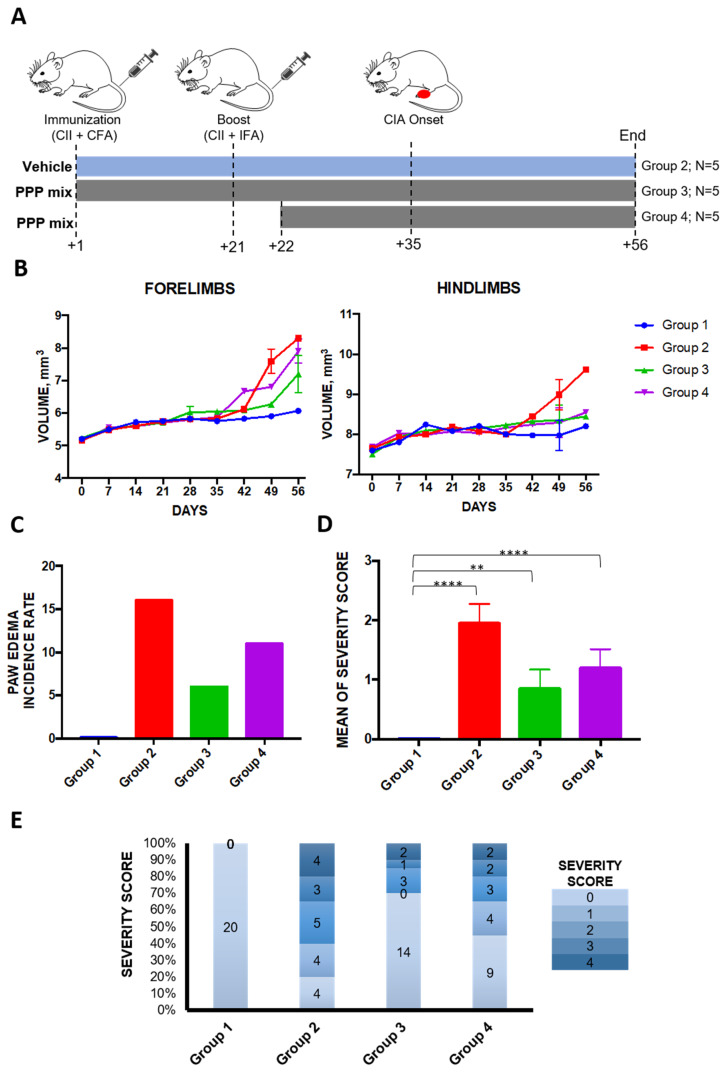
Treatment with PPP (propolis, pomegranate, and grape pomace) mixture ameliorates the pathology of collagen-induced arthritis (CIA). (**A**) Timetable of CIA induction and treatment strategies in type II collagen (CII)-immunized DBA/1J mice. All mice were sacrificed on day 56 post immunization. (**B**) The average forepaw and hindpaw volumes were measured in each experimental group. (**C**) Incidence of rheumatoid arthritis (RA) (number of paws showing clinical sympthoms). (**D**) Severity score observed in each experimental group (mean ± SEM). (**E**) Distribution of the severity scores among the experimental groups. Data are from n = 5 mice/group (n = 20 paws/group). ** *p* < 0.01; **** *p* < 0.0001, as calculated by one-way ANOVA. CII = collagen II; CFA = Complete Freund Adjuvant; IFA = Incomplete Freund Adjuvant.

To verify if a herbal preparation consisting of propolis, pomegranate peel, and grape pomace extracts (4:1:1) (hereafter PPP mixture) could have a therapeutic potential in inflammation, CIA mice were treated using two different treatment schedules (Figure 3a).

Both strategies reduced the disease severity, both when administered early at the induction of arthritis (Group 3) and when administered after CIA establishment (Group 4).

More specifically, the treatment with the PPP mixture ameliorated paw swelling and lowered the incidence (number of affected paws) and symptoms (severity score) in CIA mice (Figure 3b–d).

The early treatment with PPP mixture reduced the number of affected paws by 60% with respect to CIA-induced mice (6/20 in Group 3 vs. 16/20 in Group 2). A 30% reduction in affected paws (11/20 affected paws) was instead observed when treatment started 30 days after the CIA induction, when the pathology was well established. In the treated groups, there was also a significant reduction in the severity score and the associated inflammatory state. Indeed, the mean severity score was 1.95 in collagen-induced mice (Group 2), 0.85 in early PPP-treated mice (Group 3), and 1.2 in mice treated after the RA onset (Group 4). The severity score measured for each paw is reported in Figure 3e.

**Figure 4:**
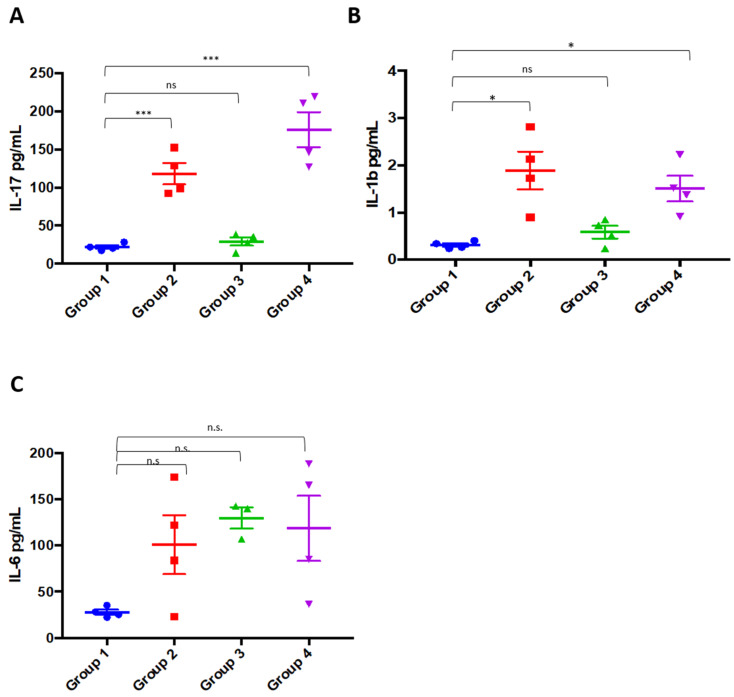
PPP (propolis, pomegranate, and grape pomace) mixture inhibits the expression of IL-17 and IL-1b. IL-17 (**A**), IL-1b (**B**), and IL-6 (**C**) levels measured in each sample of the indicated experimental groups. Symbols refer to the cytokine levels measured in each sample; horizontal lines indicate mean values ± SEM for each group. * *p* < 0.05; *** *p* < 0.001; ns/n.s. = not significant, as calculated using one-way ANOVA.

Proinflammatory cytokines, and in particular IL-17, IL-6, and IL-1b, are implicated in the pathogenesis of RA. Recent in vivo animal models and in vitro human studies demonstrated that proinflammatory cytokines play a crucial role and IL-17 can be considered a decisive mediator in the pathogenesis of RA [37]. The ELISA results confirmed a significant upregulation of IL-17 and IL-1b in collagen-induced mice (Group 2); a similar trend was also observed for IL-6. Hence, we asked whether the treatment with PPP mixture inhibited the expression of these proinflammatory factors in vivo. The early administration of the PPP mixture (Group 3) prevents IL-17 and IL-1b increases induced by the collagen II administration, while it does not affect IL-6 levels (Figure 4). The treatment with PPP after the RA onset in mice (Group 4) does not restore the cytokines’ profile, suggesting that the treatment with PPP exerts major protective effects on the RA onset (by limiting IL-17 and IL-17–triggering cytokine IL-1b production), while it only exhibits a partial therapeutic effect. Previous studies published in the literature on the efficacy of plant extracts in reducing the onset and progression of RA in a CIA model reported significant effects with a dosage of 200 mg/kg [38,39] of tested extract, and in other cases up to 500 mg/kg [40]. Compared to these results, the PPP mixture, exerting its action at dose 150 mg/kg, can be considered a promising potential agent for managing RA.

## 3. Materials and Methods

### 3.1. Chemicals

The HPLC-grade ethanol, acetonitrile, and formic acid were purchased from VWR International srl (Milano, Italy). The HPLC-grade water (18 mW) was prepared by a Milli-Q purification system (Millipore Co., Bedford, MA, USA). The quercetin reference standard was purchased from Sigma-Aldrich S.p.a. (Milano, Italy). The alpinetin, caffeic acid prenyl ester, luteolin, pinobanksin 5-methyl ether, and pinobanksin 3-*O*-acetate used as reference standards were isolated from the propolis extract by size exclusion column chromatography (Sephadex LH-20, 5 × 100 cm, flow rate 1.0 mL/min) eluting with methanol, followed by RP-HPLC using a C_18_ μ-Bondapak column—30 cm × 7.8 mm, 10 μm (Waters, Milano, Italy)—at a flow rate of 2.0 mL/min using a mixture of MeOH−H_2_O as the eluent.

### 3.2. Materials

The propolis samples were provided during 2016 by Società Agricola Artemide snc (Pietradefusi, Avellino, Campania, Italy). The samples were collected in four different rural areas located in the Avellino (AV) and Benevento (BN) provinces (Pietradefusi (AV), Melito Irpino (AV), Melizzano (BN), Vallata (AV)). The pomegranates and Aglianico grape pomace (residue from the first grape processing, formed from stalks, peels, and grape seeds) were collected in the rural area of Ariano Irpino (AV). All the samples were frozen, ground, and homogenized prior to beginnning the extraction procedures.

### 3.3. Extraction

#### 3.3.1. Preliminary Laboratory Scale Investigation on Propolis Samples

The comparison of four different propolis samples was carried out using 5 g in each of the extraction procedures. The extraction solvent was 70% ethanol in all cases. The extraction was performed with a 320 W Ultrasonic bath (Branson 2510E-MTH, Bransonic^®^, Milano, Italy). The amount of solvent used was 10:1 (*v*/*w*). The sample was placed in an Erlenmayer flask with the corresponding amount of solvent and was treated with ultrasound at 25 °C for 30 min. Each extract was evaporated in vacuo to dryness and lyophilized until at a constant weight. The total amounts of the obtained extracts were 2.15, 2.20, 2.25, and 2.32 g for Pietradefusi, Melito Irpino, Melizzano, and Vallata, respectively.

#### 3.3.2. Scale up Extract Preparation

The three natural products, Pietradefusi propolis (400 g), pomegranate peel (400 g), and Aglianico grape pomace (5 kg), used in the biological study were obtained at BioGem (Ariano Irpino) using the Naviglio^®^ extractor (EXNA0020, Napoli, Italy); Milli-Q water was used as solvent for the extraction of grape pomace, while propolis and pomegranate peel were extracted with 70% ethanol solution. The Naviglio^®^ extraction was carried out by subjecting the sample to a total of 30 programmed cycles of pressure (with a maximum pressure of 10 bars), applied to the liquid phase in contact with the propolis over a period of 3 h. The amount of solvent used was 5:1 (*v/w*). The number of hits in the dynamic phase (nd) was 12; the dynamic operative phase (td) and the static operative phase (ts) were performed for 2 min each. The extracts obtained were freeze-dried using a Stellar Millrock ST8S5-l lyophilizer (Terni, Italy) to obtain 100 g of propolis, 50 g of pomegranate peel, and 250 g of grape pomace extracts. The pietradefusi propolis extract obtained by using the Naviglio^®^ extractor (Napoli, Italy) was compared to that prepared with ultrasonic extraction by LC-MS analyses (see Section 3.4), obtaining an identical chemical profile (data not shown).

### 3.4. LC-MS Analyses

#### 3.4.1. Quali-Quantitative Analyses of Phenols in Propolis Extracts

All the propolis extracts were dissolved in MeOH at a final concentration of 2.0 mg/mL, then centrifuged for 10 min at 1145× *g*. The supernatants (20 μL injection volume) were subjected to chemical analyses by a HPLC-PDA/UV-ESI-MS/MS system composed of a Surveyor autosampler, a Surveyor LC pump, a Surveyor PDA/UV detector, and an LCQ Advantage ESI-ion trap mass spectrometer (ThermoFinnigan, San Jose, CA, USA) equipped with Xcalibur 3.1 software. A Luna (C-18) column, 4.6 × 150 mm, 5 μm (Phenomenex, Bologna, Italy) was used for LC-MS analyses, eluting with a mixture of acetonitrile (solvent A) and formic acid in water 0.1% *v/v* (solvent B) and using the following solvent gradient in the ESI negative ion mode: 0–5 min, 5–10% A; 5–10 min, 10–15% A; 10–15 min, 15–20% A; 15–20 min, 20–25% A; 20–40 min, 25–30% A; 40–45 min, 30–35% A; 45–55 min, 35–40% A; 55–65 min, 40–60% A; 65–75 min, 60–90% A. In the positive ion mode, the following solvent gradient was used: 0–5 min, 5–10% A; 5–10 min, 10–15% A; 10–15 min, 15–20% A; 15–20 min, 20–25% A; 20–40 min, 25–30% A; 40–45 min, 30–35% A; 45–55 min, 35–40% A; 55–65 min, 40–60% A; 65–75 min, 60–75% A. Analyses were performed at a flow rate of 0.8 mL/min, with a splitting system of 2:8 to the MS detector (160 μL/min) and PDA detector (640 μL/min), respectively. PDA/UV data were recorded at 200–600 nm using 254, 280, and 325 nm as preferential channels. The ESI interface was used both in the negative and positive ion modes, with a scan range of *m/z* 150–2000, using N_2_ as a sheath and auxiliary gas. The ionization conditions used in the negative ion mode were previously reported [41], while in the positive ion mode they were optimized as follows: capillary temperature, 250 °C; source voltage, 4.50 kV; capillary voltage, 29.0 V; tube lens offset, 50 V; sheath gas flow rate, 60.00 arbitrary units; auxiliary gas flow rate, 3.00 arbitrary units. The ESI-MS/MS experiments were performed using 35.0% normalized collision energy.

Quantitative analyses of the main phenolics detected in all the analysed propolis extracts were performed by different calibration curves using the following standards: (a) positive ion mode: quercetin (concentration range 0.007–1.0 mg/mL), luteolin (concentration range 0.003–0.500 mg/mL), pinobanksin 5-methyl ether (concentration range 0.003–0.500 mg/mL), and alpinetin (concentration range 0.001–0.500 mg/mL), to quantify flavonols, flavones, dihydroflavonols, and flavanone, respectively; (b) negative ion mode: caffeic acid prenyl ester (concentration range 0.0025–1.0 mg/mL) and pinobanksin 3-*O*-acetate (concentration range 0.003–0.5 mg/mL) to quantify caffeic acid and pinobanksin derivates, respectively. The stock solutions (1 mg/mL) were prepared for each standard and at least four different concentrations obtained by serial dilution were injected in triplicate. Each calibration curve was generated by using concentration (mg/mL) with respect to the area obtained from the integration of the MS molecular ion of each standard ([M − H]^−^ in the negative ion mode and [M + H]^+^ in the positive mode). The relation between the variables was analyzed using linear simple correlation. All the propolis extracts were injected in triplicate and the amount of each constituent of propolis extract was calculated by using a GraphPad Software Prism 6.0 and finally expressed as g/100 g of raw propolis.

#### 3.4.2. Chemical Characterization of Pomegranate Peel and Grape Pomace

The pomegranate extract was dissolved in MeOH (2.5 mg/mL), centrifuged for 10 min at 1145× *g* and injected into the HPLC-PDA/UV-ESI-MS/MS (20 μL injection volume). A Synergi Fusion-RP column, 4.6 × 150 mm, 4 μm particle size (Phenomenex, Bologna, Italy) was used, eluting at a flow rate of 0.8 mL/min with methanol (solvent A) and formic acid in water 0.1% *v/v* (solvent B), developing a linear gradient of increasing 5% to 55% A within 50 min. The ESI interface was used in the negative ion mode (scan range of *m/z* 150–2000) with the same ionization conditions utilized for the propolis extract analyses. The PDA/UV data were registered in a wavelength range of 200–600 nm, using 254, 280, and 325 nm as preferential channels.

The grape pomace was dissolved in formic acid in water 0.1% *v/v* and analyzed by a LTQ-Orbitrap XL mass spectrometer (Thermo Fisher Scientific Inc., Bremen, Germany). The elution was performed on a Luna C-18 column 100 × 2 mm, 2.5 µm (Phenomenex, Bologna, Italy) at a flow rate of 0.2 mL/min (10 μL injection volume), using a mixture of formic acid in water 0.1% *v/v* (solvent A) and acetonitrile (solvent B) and had the following linear solvent gradient: 5% to 95% of B in 40 min. The ESI interface was used in the positive ion mode (scan range of *m/z* 200–1000), using N_2_ as a sheath and auxiliary gas. The ionization conditions were optimized as follows: capillary temperature, 275 °C; source voltage, 5.0 kV; capillary voltage, 35.0 V; tube lens offset, 100 V; sheath gas flow rate, 30.00 arbitrary units; auxiliary gas flow rate, 10.00 arbitrary units. The ESI-MS/MS experiments were performed using a 30.0% normalized collision energy.

### 3.5. Collagen-Induced Arthritis Assay

The 8–12-weeks-old female DBA/1j mice were purchased from Charles River Laboratories. The mice were housed inside individually ventilated cages (IVC) of polisulfone, keeping the temperature and humidity constant. All the animal studies were conducted in accordance with ethics approval obtained from the Italian Ministry of Health (D.Lgs. 26/2014), and all the experiments were in accordance with the European guidelines for the care and use of laboratory animals (Directive 2010/63/EU). Food and drinking water were supplied ad libitum. Each mouse was offered daily a complete pellet diet (GLP 4RF21, Mucedola s.r.l, Milano, Italy) throughout the study. The CIA was elicited by immunization with collagen CII (C9301-5MG, Sigma Aldrich, Milano, Italy) emulsified in Complete Freund’s Adjuvant (CFA, F5881-10ML, Sigma Aldrich, Milano, Italy) and Incomplete Freund’s Adjuvant (IFA, F5506-10ML, Sigma Aldrich, Milano, Italy) by intradermal injections (50 µL/mouse). The emulsion was prepared with equal volumes of CII and CFA or IFA, considering 50 μL of emulsion per mouse. For immunization, an intradermal injection was made at about 1.5 cm distal from the base of the tail. An amount of 50 µL of CII+CFA (day 1) or CII+IFA (day 29, booster injection) emulsion was slowly injected.

The DBA/1J mice were divided into four experimental groups (5 mice/group): Group 1, non-induced control group, treated with vehicle (5% TWEEN^®^ 80 in H_2_O, 10 mL/kg); Group 2, CIA-induced positive control group (treated with 50 μL of bovine type II collagen emulsified in CFA on day +1 and +21); Group 3, collagen-induced mice treated with the PPP mixture (propolis (100 mg/kg) + pomegranate extract (25 mg/kg) + pomace extract (25 mg/kg)), dissolved in vehicle, from the day +1; Group 4, treated with the PPP mixture from day +22. The water or mixture was orally administered by gavage.

The evaluation of the onset of the disease was evaluated weekly by observing the characteristic symptoms of the disease on the animals’ paws and assigning a degree of severity (score) as described previously and reported in Table 6.

**Figure 5:**
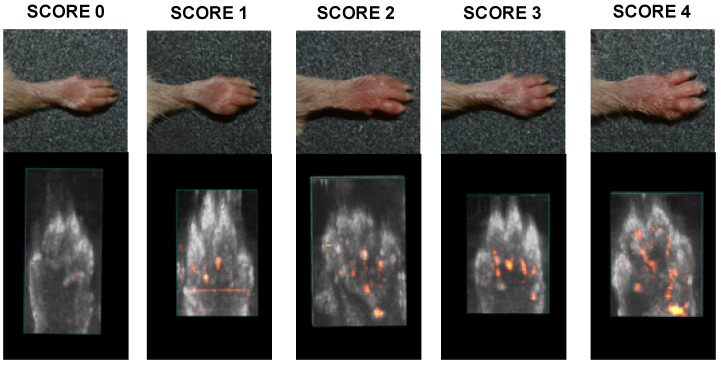
Arthritic paws with severity scores and associated Pulsed Wave (PW) inflammatory status.

At the end of the study period (36 days), the score was calculated for each paw of each animal (5 group animals/20 paws per group), and a qualitative assessment of the associated inflammatory state was also performed using Pulsed Wave (PW) doppler echography (Figure 5).

Serum was collected from untreated and treated animals at different days +36 and kept frozen at −20 °C until analysis. A commercially available ELISA kit was performed according to the manufacturer’s instructions for the analysis of IL-17 (E-EL-M0047), IL-1b (E-ELM0037), and IL-6 (E-EL-M0044) (Elabscience, Verona, Italy). The cytokine concentration has been determined by interpolation with the 4-PL standard curve, using the GraphPad Prism v7 software (GraphPad, Arezzo, Italia).

### 3.6. Statistical Analysis

Statistical analyses have been performed by GraphPad Prism v7 software (GraphPad, Arezzo, Italia). The ELISA data are expressed as mean (SEM). A Kruskal–Wallis test followed by Dunn’s post test was used for the statistical analyses of multiple comparisons.

## 4. Conclusions

Our study highlights the impact of the PPP mixture on RA onset and progression. By using in vivo CIA mouse model, we confirmed that treatment with PPP alleviated the severity of clinical symptoms. Furthermore, an early PPP treatment was associated with a reduction in the serum levels of IL-17 and IL-17–triggering cytokines. Therefore, it is plausible that PPP treatment, by preventing the IL-17 and IL-1b increase, could improve/expand the current therapeutic options for RA patients.

Moreover, our results confirmed the anti-inflammatory activity of a traditional rural preparation from internal Campanian areas based on propolis and grape pomace. The high polyphenols content of the PPP herbal preparation is strictly linked to its anti-inflammatory activity and could be considered as a starting material to develop a new valuable herbal preparation against RA. Currently, the pharmacological approaches to treat RA patients use non-steroidal, anti-inflammatory, or disease-modifying antirheumatic drugs. The side effects of these drugs can be overcome by the identification of natural products capable of relieving the symptoms of RA.

## Figures and Tables

**Table 1:** Chromatographic (*t*_R_ = retention time), UV, and negative ion mode electrospray ionization—tandem mass spectrometry (ESI-MS/MS) data of the constituents identified in Italian propolis extracts: A, Pietradefusi; B, Melito Irpino; C, Melizzano; D, Vallata.

Peak *	Compound	*t*_R_ (min)	M	[M − H]^−^	[M + HCOO]^−^	ESI-MS/MS Product Ions **	UV (λ_max_)	Extract
**1**	Caffeic acid	15.1	180	179	-	**135**	243, 324	A-D
**4**	Pinobanksin 5-methyl ether	34.1	286	285	331	**267**, 253, 239, 179, 139	236, 287	A-D
**5**	Quercetin 3-methyl ether	36.1	316	315	-	**300**, 271, 228	256, 357	A-D
**6**	Chrysin 5-methyl ether	38.6	268	**267**	313	252, 224	264, 319	A-D
**7**	Apigenin	41.2	270	**269**	315	225, 151, 117	268, 335	A-D
**8**	Pinobanksin	42.4	272	**271**	317	253, 243, 165, 107	236, 291	A-D
**9**	Kaempferol	43.7	286	**285**	331	257, 241	269, 364	A-D
**12**	Isorhamnetin	45.2	316	315	361	**300**, 151	255, 370	A-D
**13**	Luteolin 3′-methyl ether	47.2	300	299	345	**284**, 256, 151	267, 351	A-D
**14**	Quercetin dimethyl ether	48.4	330	329	375	**314**, 299, 285, 243	255, 356	A-D
**15**	Galangin 5-methyl ether	50.3	284	283	329	268, 239	260, 300, 351	A-D
**16**	Pinobanksin 5-methyl ether-3-*O*-acetate	51.2	328	327	373	**285**, 267, 252, 224	309	A-D
**17**	Luteolin methyl ether	52.7	300	299	-	**284**	268, 347	A-D
**18**	Quercetin 7-methyl ether	53.1	316	315	361	**300**, 271, 256, 193, 165	256, 368	A-D
**19**	Quercetin dimethyl ether	56.0	330	329	-	**314**, 299	256, 357	A-D
**20**	Caffeic acid prenyl ester	59.2	248	247	293	**179**, 135	245, 326	A-D
**21**	Chrysin	59.7	254	253	-	**254**, 209	268, 321	A-D
**23**	Caffeic acid benzyl ester	60.3	270	269	-	**178**, 134	295, 320	A-D
**24**	Caffeic acid prenyl ester	60.6	248	247	293	**179**, 203, 135	245, 327	A-D
**26**	Pinocembrin	61.7	256	255	300	**300**, 213, 187, 151, 145	237, 289	A-C
**27**	Galangin	62.1	270	269	315	**269**, 227, 197	266, 359	A-C
**30**	Caffeic acid phenylethyl ester (CAPE)	62.9	284	283	329	**179**, 135	301, 326	A-C
**31**	Pinobanksin 3-*O*-acetate	63.3	314	313	359	271, **253**, 209	237, 293	A-C
**32**	Methoxychrysin	64.0	284	283	329	268, 239, 211	266, 335	A-D
**33**	Caffeic acid cinnamyl ester	65.9	296	295	341	251, 211, **178**, 134	248, 301, 313	A-D
**34**	Pinobanksin 3-*O*-propionate	67.3	328	327	373	271, **253**	293	A-D
**35**	Pinobanksin 3-*O*-butyrate	70.3	342	341	-	271, **253**	248, 292	A-D
**37**	Pinobanksin 3-*O*-pentanoate	72.5	356	355	401	271, **253**	292	A-D
**38**	Pinobanksin 3-*O*-hexanoate	73.9	370	369	-	271, **253**	292	A-D

* Compounds are listed in order of increasing *t*_R_ and numbers correspond with the peak numbers in Figure 1. ** Product ions are generated by the fragmentation of [M − H]^−^; the base ion peaks generated in the ESI-MS/MS experiments are shown in bold.

**Table 2:** Chromatographic (*t*_R_ = retention time), UV, and positive ion mode ESI-MS/MS data of the constituents identified in Italian propolis extracts: A, Pietradefusi; B, Melito Irpino; C, Melizzano; D, Vallata.

Peak *	Compound	*t*_R_ (min)	[M + H]^+^	ESI-MS/MS Product Ions **	UV (λ_max_)	Extract
**2**	Luteolin methyl ether	29.1	301	286	259, 358	C
**3**	Quercetin dimethyl ether	30.3	331	316, 301	252, 362	A-D
**4**	Pinobanksin 5-methyl ether	33.8	287	269, **241**, 91	288	A-D
**5**	Quercetin 3-methyl ether	35.7	317	**302**, 165, 153, 137	256, 357	A-D
**6**	Chrysin 5-methyl ether		**269**	254, 167	262, 329	A-D
**7**	Apigenin	40.7	**271**	247, 153	268, 337	A-D
**10**	Pinobanksin methyl ether	43.0	**287**	269, 241	266, 366	B-D
**11**	Alpinetin	43.6	271	167, 131	268, 365	A, D
**12**	Isorhamnetin	44.6	317	**302**, 285, 261, 257	254, 370	A, C
**13**	Luteolin 3′-methyl ether	46.8	301	**286**	267, 350	A-D
**14**	Quercetin dimethyl ether	48.0	331	**316**, 301, 299	253, 355	A-D
**15**	Galangin 5-methyl ether	50. 0	285	**270**, 167	260, 352	A-D
**16**	Pinobanksin 5-methyl ether-3-*O*-acetate	51.2	329	**287**, 269, 241, 167	289, 329	A-D
**17**	Luteolin methyl ether	52.2	**301**	286	266, 348	A-D
**18**	Quercetin 7-methyl ether	52.6	**317**	302, 299, 271, 243, 179, 167	256, 370	A-D
**19**	Quercetin dimethyl ether	55.6	331	**316**, 299	256, 356	A-D
**21**	Chrysin	59.3	**255**	209, 153, 129	268, 314	A-D
**22**	Flavonoid aglycon methyl ether	60.1	**285**	270	245, 327	B-D
**25**	Flavonoid aglycon methyl ether	61.0	**285**	270	272, 318	A-D
**26**	Pinocembrin	61.3	257	215, 153, **131**, 103	290	A-D
**27**	Galangin	61.7	**271**	165, 153	246, 327	A-D
**28**	Luteolin methyl ether	62.1	**301**	286	268, 362	A-D
**29**	Quercetin dimethyl ether	62.8	331	**316**, 299	296, 326	A-D
**31**	Pinobanksin 3-*O*-acetate	63.0	315	**273**, 255, 227, 153	296, 326	A-D
**32**	Methoxychrysin	63.7	285	**270**, 242	293	A-D
**36**	Chrysin 5-methyl ether	70.8	**269**	254, 167	289	A-D

* Compounds are listed in order of increasing *t*_R_ and numbers correspond with the peak numbers in Figure 2. ** Product ions are generated by the fragmentation of [M + H]^+^; the base ion peaks generated in the ESI-MS/MS experiments are shown in bold.

**Table 3:** Quantitative amount (g/100 g ± standard deviation of raw propolis) of constituents detected in the propolis extracts.

Peak	Compound	Pietradefusi	Melito Irpino	Melizzano	Vallata
**1**	Caffeic acid	0.308 ± 0.01	0.273 ± 0.01	0.150 ± 0.03	0.203 ± 0.01
**3**	Quercetin dimethyl ether	0.014 ± 0.00	0.010 ± 0.00	0.005 ± 0.00	Trace *
**4**	Pinobanksin 5-methyl ether	0.971 ± 0.01	0.557 ± 0.05	0.354 ± 0.03	0.219 ± 0.01
**5**	Quercetin 3-methyl ether	0.843 ± 0.04	0.288 ± 0.01	0.252 ± 0.01	0.092 ± 0.00
**6**	Chrysin 5-methyl ether	0.612 ± 0.01	0.309 ± 0.03	0.260 ± 0.01	0.103 ± 0.01
**7**	Apigenin	0.378 ± 0.01	0.240 ± 0.02	0.179 ± 0.09	0.103 ± 0.02
**8**	Pinobanksin	1.226 ± 0.12	0.620 ± 0.00	0.542 ± 0.06	0.477 ± 0.03
**9**	Kaempferol	0.038 ± 0.00	0.021 ± 0.00	0.012 ± 0.00	0.031 ± 0.00
**10**	Pinobanksin methyl ether	0.126 ± 0.01	0.149 ± 0.02	0.071 ± 0.01	0.089 ± 0.01
**11**	Alpinetin	0.221 ± 0.00	0.127 ± 0.00	0.069 ± 0.01	0.053 ± 0.00
**12**	Isorhamnetin	0.267 ± 0.01	0.141 ± 0.01	0.020 ± 0.00	0.011 ± 0.00
**13**	Luteolin 3′-methyl ether	0.656 ± 0.00	0.361 ± 0.02	0.299 ± 0.02	0.170 ± 0.01
**14**	Quercetin dimethyl ether	0.791 ± 0.03	0.272 ± 0.01	0.281 ± 0.01	0.051 ± 0.00
**15**	Galangin 5-methyl ether	1.217 ± 0.00	0.653 ± 0.00	0.494 ± 0.00	0.265 ± 0.00
**16**	Pinobanksin 5-methyl ether-3-*O*-acetate	0.030 ± 0.00	0.020 ± 0.00	0.013 ± 0.00	0.012 ± 0.00
**17**	Luteolin methyl ether	0.479 ± 0.02	0.279 ± 0.03	0.143 ± 0.01	0.076 ± 0.08
**18**	Quercetin 7-methyl ether	0.374 ± 0.02	0.101 ± 0.00	0.051 ± 0.00	0.047 ± 0.00
**19**	Quercetin dimethyl ether	1.981 ± 0.13	0.704 ± 0.05	0.754 ± 0.03	0.278 ± 0.01
**20**	Caffeic acid prenyl ester I	3.930 ± 0.13	3.682 ± 0.05	1.779 ± 0.36	2.973 ± 0.12
**21**	Chrysin	4.535 ± 0.06	2.743 ± 0.08	2.267 ± 0.14	1.452 ± 0.08
**22**	Flavonoid aglycon methyl ether	0.323 ± 0.00	0.146 ± 0.01	0.097 ± 0.01	0.046 ± 0.01
**23**	Caffeic acid benzyl ester	3.220 ± 028	2.186 ± 0.11	0.914 ± 0.13	1.990 ± 0.14
**24**	Caffeic acid prenyl ester II	3.695 ± 0.02	2.750 ± 0.09	2.032 ± 0.28	1.574 ± 0.03
**25**	Flavonoid aglycon methyl ether	0.676 ± 0.03	0.392 ± 0.01	0.256 ± 0.04	0.040 ± 0.00
**26**	Pinocembrin	0.594 ± 0.02	0.387± 0.01	0.203 ± 0.02	0.140± 0.08
**27**	Galangin	0.983 ± 0.01	0.662 ± 0.00	0.351 ± 0.01	0.202 ± 0.03
**28**	Luteolin methyl ether	0.295 ± 0.01	0.389 ± 0.02	0.300 ± 0.03	0.168 ± 0.00
**29**	Quercetin dimethyl ether	0.139 ± 0.00	0.181 ± 0.00	0.466 ± 0.00	0.016 ± 0.00
**30**	Caffeic acid phenylethyl ester (CAPE)	4.100 ± 0.26	2.787 ± 0.07	1.811 ± 0.30	2.088 ± 0.13
**31**	Pinobanksin 3-*O*-acetate	1.415 ± 0.09	1.139 ± 0.02	0.747 ± 0.13	0.925 ± 0.06
**32**	Methoxychrysin	1.030 ± 0.01	0.590 ± 0.01	0.553 ± 0.05	0.249 ± 0.02
**33**	Caffeic acid cinnamyl ester	1.08 ± 0.07	0.715 ± 0.03	0.438 ± 0.09	0.910 ± 0.09
**34**	Pinobanksin 3-*O*-propionate	0.471 ± 0.03	0.249 ± 0.01	0.255 ± 0.04	0.177 ± 0.02
**35**	Pinobanksin 3-*O*-butyrate	0.292 ± 0.02	0.176 ± 0.01	0.143 ± 0.03	0.137 ± 0.01
**36**	Chrysin 5-methyl ether	4.450 ± 0.13	1.642 ± 0.05	1.884 ± 0.10	0.705 ± 0.70
**37**	Pinobanksin 3-*O*-pentanoate	0.509 ± 0.04	0.289 ± 0.01	0.261 ± 0.04	0.231 ± 0.02
**38**	Pinobanksin 3-*O*-hexanoate	1.415 ± 0.00	0.012 ± 0.00	0.014 ± 0.00	0.009 ± 0.00
	Total flavonoids	22.85 ± 0.86	12.99 ± 0.49	10.92 ± 0.92	5.800 ± 1.23
	Total phenolic acids	16.33 ± 0.70	12.40 ± 0.37	7.124 ± 0.92	9.739 ± 0.53
	Total phenols	39.18 ± 1.56	25.39 ± 0.86	18.04 ± 1.84	15.54 ± 1.76

* < Limit of detection.

**Table 4:** Main components of the pomegranate peel extract.

Compound	[M − H]^−^	ESI-MS/MS Product Ions *	UV (λ_max_)
**Pomegranate**			
Ellagitannins			
Ellagic acid	301	229, 173	254, 368
Ellagic acid pentoside	433	**301**, 285	254, 363
Ellagic acid hexoside	463	**301**	253, 362
HHDP-hexoside	481	**301**, 275	237
Galloyl-HHDP-hexose	633	463, **301**, 275	235, 257
Ellagic acid derivative	799	781, **479**, 301	235, 259
Galloyl-HHDP-DHHDP-hexose (granatin B)	952	**933**, 915, 613, 445, 301	237, 261
Castalagin derivative	965	**933**, 915, 781, 631, 445, 301	242, 269
Gallagyl derivatives			
Gallagyl-hexose (punicalin isomer I)	781	721, **601**, 575, 449, 299	223, 259, 376
Gallagyl-hexose (punicalin isomer II)	781	721, **601**, 575, 449, 299	234, 259, 380
HHDP-gallagyl-hexose (punicalagin isomer I)	541 **	781, 601, 575, **301**, 275	235, 259, 380
HHDP-gallagyl-hexose (punicalagin isomer II)	541 **	781, 601, 575, **301**, 275	236, 258, 379

DHHDP = dehydrohexahydroxydiphenic acid; HHDP = bis-hexahydroxydiphenoyl hexoside. * Base ion peaks are shown in bold. ** Doubly charged ion species [M − 2H]^2−^ corresponding to 1084 u.

**Table 5:** Main components of the Aglianico grape pomace extract.

Compound	HR-[M + H]^+^/[M]^+^	HR-ESI-MS/MS Product Ions	Error (ppm)
Phenolics			
Resveratrol	229.0858	211, 135	−0.43
Catechin/epicatechin	291.0860	165, 139, 123	−1.03
Quercetin	303.0499	257, 229, 165, 137	−0.01
Cyanidin 3-*O*-glucoside	449.1070 *	287	−1.78
Delphinidin 3-*O*-glucoside	465.1022 *	303	−1.07
Petunidin 3-*O*-glucoside	479.1176 *	317	−1.67
Quercetin 3-*O*-glucuronide	479.0812	303	−1.67
Malvidin 3-*O*-glucoside	493.1329 *	331	−2.23
Myricetin 3-*O*-glucuronide	495.0751	319	−3.64
Laricitricin 3-*O*-glucoside	495.1127 *	333	−1.21
Syringetin 3-*O*-glucoside	509.1278	347	−2.36
Vitisin B (malvidin-3-O-glucoside acetaldehyde)	517.1331 *	355	−1.74
Malvidin 3-*O*-acetylglucoside	535.1430 *	331	−2.99
Vitisin A (malvidin-3-*O*-glucoside pyruvate)	561.1223 *	399	−2.85
Procyanidin dimer	579.1476	427, 291	−3.63
Procyanidin dimer	595.1439	443, 291	−1.18
Malvidin 3-*O*-*p*-coumaroylglucoside	639.1699 *	331	−1.41
Malvidin 3-*O*-glucoside-ethyl-(epi)catechin	809.2177 *	647, 519, 357	−1.41
Procyanidin trimer	867.2130	579, 427	−0.11
Malvidin 3-*O*-*p*-coumaroylglucoside-ethyl-(epi)catechin	955.2630 *	665, 357	−2.62

HR = high-resolution. * The molecular ion is represented by [M]^+^.

**Table 6:** Scoring system for the subjective evaluation of arthritis severity.

Severity Score	Degree of Inflammation
0	No evidence of erythema and swelling
1	Erythema and mild swelling confined to the tarsals or ankle joint
2	Erythema and mild swelling extending from the ankle to the tarsals
3	Erythema and moderate swelling extending from the ankle to metatarsal joints
4	Erythema and severe swelling encompass the ankle, foot and digits, or ankylosis of the limb
